# Mycophenolate mofetil therapy for steroid-resistant IgA nephropathy with the nephrotic syndrome in children

**DOI:** 10.1007/s00467-014-3041-y

**Published:** 2015-03-15

**Authors:** Zhijuan Kang, Zhihui Li, Cuirong Duan, Tianhui Wu, Mai Xun, Yunfeng Ding, Yi Zhang, Liang Zhang, Yan Yin

**Affiliations:** 1 Hunan Institute for Pediatric Research & Department of Nephrology, Hunan Children’s Hospital, 86 Ziyuan Road, Changsha, Hunan 410007 People’s Republic of China; 2 Academy of Pediatrics of University of South China, 86 Ziyuan Road, Changsha, Hunan 410007 People’s Republic of China

**Keywords:** IgA nephropathy, Nephrotic syndrome, Corticosteroids, Mycophenolate mofetil, Children

## Abstract

**Background:**

Immunoglobulin A nephropathy (IgAN) presents as nephrotic syndrome (NS) relatively rarely, and the current treatment experience of IgAN patients with NS is mostly with adults. The objective of our study was to investigate the efficacy of corticosteroids and mycophenolate mofetil (MMF) in treating childhood immunoglobulin A nephropathy (IgAN) with nephrotic syndrome.

**Methods:**

A total of 58 children (39 boys and 19 girls) diagnosed with nephrotic syndrome and primary IgAN were enrolled in the study. All the patients were administered prednisone 2 mg/kg per day for 8 weeks. Steroid-resistant patients were treated with the combined use of MMF (dose of 20 ~ 30 mg/kg per day) and prednisone for 6–12 months. The prednisone dose was reduced stepwise during the combined treatment.

**Results:**

Of the 58 children, 14 were steroid-sensitive (M, S, and T variants of the Oxford classification were 0 in most children), and 44 cases who presented serious pathological damage to the kidney were steroid-resistant. The estimated glomerular filtration rate (eGFR) of the steroid-resistant children (86.69 ± 26.85 ml/min/1.73 m^2^) was significantly lower (*P* < 0.05) than that of the steroid-sensitive children (106.89 ± 26.94 ml/min/1.73 m^2^). After 4 months of combined MMF treatment in 33 steroid-resistant children, complete remission of proteinuria was found in 21 cases, partial remission of proteinuria in 6 cases, and no response was found in 6 cases. Except for the T variant, other variants of the Oxford classification, including M, E, and S morphological variables, was not significantly different among patients complete remission, those with partial remission, and those with no response. The eGFR of children with complete remission of proteinuria (100.04 ± 18.47 ml/min/1.73 m^2^), that of those with partial remission (92.24 ± 27.63 ml/min/1.73 m^2^), and that of those with no response (72.17 ± 27.55 ml/min/1.73 m^2^) were significantly different (*P* < 0.05).

**Conclusion:**

Corticosteroid therapy showed satisfactory efficacy in IgAN children with nephrotic syndrome and slight pathological damage. The effect of MMF was good for steroid-resistant IgAN children, but poor for those with tubular atrophy/interstitial fibrosis and renal function impairment.

## Introduction

Immunoglobulin A nephropathy (IgAN) is the most common form of glomerulonephritis (GN) worldwide, comprising 45 % of all primary GN cases in China too [1, 2]. Many studies report that 30–40 % of adult patients with IgAN reach end-stage renal disease (ESRD) within 20 years of apparent disease onset [2–4]. A study of 241 Japanese pediatric patients revealed that 11 % of the patients exhibited end-stage renal failure (ESRF) within 15 years [5]. The degree of proteinuria is one of the strongest predictors of outcome in IgAN [1, 6, 7], and the risk of renal failure increases with higher proteinuria levels. In contrast, lowering proteinuria markedly decreases the risk, regardless of whether the initial proteinuria is mild or within the nephrotic range [1, 7].

In most cases, the initial manifestations of IgAN are recurrent episodes of gross hematuria that usually occur after upper respiratory tract infections and asymptomatic microscopic hematuria, with or without mild proteinuria. IgAN presents as nephrotic syndrome (NS) relatively rarely, having a prevalence rate that varies from 5 to 20 % in adults [8–16]. A multi-center study by Kim et al. reported that 10.2 % of children with IgAN had clinical features of NS [17]. On the other hand, a multi-center study in China [18] reported that 30.6 % of children with IgAN had clinical manifestations of NS. Steroid therapy is effective if the IgAN patients with NS show pathological changes similar to minimal lesions [14, 19–21], but it is less effective if they have the pathological changes with diffuse mesangial proliferation [22]. The current treatment experience of IgAN patients with NS is mostly with adults.

How do IgAN children with clinical manifestation of NS respond to corticosteroid therapy? Is steroid treatment combined with immunosuppressant therapy effective in steroid-resistant children? How are pathological changes and lesions in IgAN related to therapeutic efficacy? These are still open questions. In order to answer these questions we conducted a prospective clinical trial in which IgAN children with clinical manifestations of NS were treated with prednisone 2 mg/kg/day and steroid-resistant children were administered concomitant mycophenolate mofetil (MMF). The responses to the combined therapy with corticosteroid and MMF were observed to investigate the factors influencing their efficacy. This study may provide a potential treatment strategy for the treatment of IgAN children with NS.

## Patients and methods

### General study outline

This was an observational prospective, single-center trial. Patients were enrolled between December 2005 and May 2013 at Hunan Children’s Hospital, Changsha, China. The ethics committee of the hospital approved the study protocol. Consent was obtained from a parent or guardian after written and oral information had been provided.

### Patients

The inclusion criteria for the study were:

1. Nephrotic syndrome. The definition and criteria for nephrotic syndrome were the same as those used in the International Study of Kidney Disease (ISKDC) [23]: serum albumin < 2.5 g/dL, and proteinuria > 40 mg/m^2^/h
2. Immunoglobulin A nephropathy. Primary IgAN was confirmed by pathological diagnosis. The patients had definite pathological data with predominantly mesangial deposition of IgA with 2+ on immunofluorescent staining and electron-dense deposits within the mesangium detected by electron microscopy
3. Age under 16 years

Exclusion criteria were:

1. Congenital kidney disease or secondary IgA nephropathy such as Henoch–Schönlein purpura nephritis, hepatitis virus-associated glomerulonephritis
2. Chronic liver and kidney dysfunction

### Renal biopsy criteria and histological evaluation of renal biopsy specimens

Renal biopsy criteria for the study were:

1. Steroid-resistance, or frequent relapse
2. Gross hematuria, or age over 10 years with microscopic hematuria
3. Renal insufficiency
4. Chronic tonsillitis when of school age
5. Family history of renal disease

Renal pathology data included information on the number of glomeruli (biopsies containing fewer than eight glomeruli were excluded from analysis) and the presence of global or segmental sclerosis, mesangial hypercellularity, endocapillary proliferation, tubular atrophy, and interstitial fibrosis. All cases were classified using light microscopy according to the Oxford classification [24, 25], which scored four key pathological features in each specimen:

1. Mesangial hypercellularity (M) was scored as M0 if >50 % of glomeruli had more than three cells per mesangial area
2. Segmental glomerulosclerosis (S) was scored as absent (S0) or present (S1)
3. Endocapillary hypercellularity (E) was scored as absent (E0) or present (E1)
4. Tubular atrophy/interstitial fibrosis (T) was based on the ratio of tubular atrophy/interstitial fibrosis in the total interstitium and scored as T0 (0–25 %). T1 (26–50 %), T2 (>50 %)

### Data collection

Information collected about the patients included age, gender, medical history, disease duration/course, clinical symptoms, effects of corticosteroid and immunosuppressive therapy, and follow-up time. Data were obtained from laboratory tests, including 24-h urinary protein, renal ultrasound, complete blood count, serum creatinine, serum albumin, serum cholesterol, serum IgA levels, and estimated glomerular filtration rate (eGFR). The eGFR was calculated according to the Schwartz formula: creatinine clearance (ml/min/1.73 m^2^) = K × L/Pcr where L is body length (cm), Pcr plasma creatinine (μmol), K: full term, <1 year, K is 39.8, 2 ~ 12 years, K is 48.6, 13 ~ 21 years (female), K is 48.6, and 13 ~ 21 years (male), K is 61.9.

### Definitions

Patients were assumed to be steroid-sensitive if the urine protein test was negative (remission) after prednisone (2 mg/kg per day) had been orally administered for ≤ 8 weeks, whereas patients were classified as steroid-resistant if the urine protein test was not negative (no reaction) after prednisone (2 mg/kg per day) had been orally administered for > 8 weeks. Patients were assumed to be MMF-resistant if urine protein test was not negative (no reaction) after MMF (20 ~ 30 mg/kg per day) had been orally administered for 4 months.

Complete remission (CR) was defined as the absence of proteinuria (UPCR < 0.3 g/g), normalization of all biochemical findings, and no worsening of renal function. Partial remission (PR) was defined as a > 50 % reduction in proteinuria from baseline to 50 mg/kg/day. No response (NR) was defined as a < 50 % reduction in proteinuria or an increase in proteinuria. Acute kidney injury (AKI) was defined, using the KDIGO guidelines [26], as any of the following:

1. An increase in serum creatinine by ≥ 26.5 μmol/L (≥ 0.3 mg/dL) within 48 h
2. An increase in serum creatinine to ≥ 1.5 times the baseline, which is known to have occurred within the previous 7 days

### Study medications

Children enrolled in this study were administered prednisone, in which the initial dose was 2 mg/kg per day and the maximum dose was no more than 60 mg/day. Prednisone was administered as a single dose in the morning for 8 weeks. For patients in complete remission at the end of 8 weeks, prednisone was taken every other day at a dose that was two-thirds of the original 2-day dose at first, and that was reduced by 2.5–5 mg every 2 weeks until drug withdrawal. However, for those without remission at the end of 8 weeks, 20–30 mg/kg per day of MMF was divided into two equal doses and used in combination with prednisone for 6 to 12 months. The dose of prednisone was reduced by 2.5–5 mg every 2 to 4 weeks at first, maintained at the dose of 1 mg/kg per day for 8 weeks, and then reduced by 2.5–5 mg every 2 weeks until withdrawal.

### Statistical analyses

Variables with normal distributions were expressed as mean ± SD, and were compared using the *t* test. Categorical variables were expressed as percentages and compared using the Chi-squared test. Statistical significance was determined as *P* < 0.05. SPSS software (version 18.0; SPSS, Chicago, IL, USA) was used for all statistical analyses.

## Results

### Baseline characteristics

A total of 171 children were diagnosed with primary IgAN between December 2005 and May 2013 at the Hunan Children’s Hospital, China, which accounted for 5.4 % of all kidney biopsies. Among 171 children with IgAN, 122 patients were male, 49 patients were female, and their median age was 8.86 ± 3.24 years. There were 60 patients (33.7 %) with the nephrotic syndrome, 28 patients with simple hematuria and 83 patients with the other symptoms (Table 1).

**Table 1:** Baseline characteristics of patients with immunoglobulin A (IgA) nephropathy

	All patients (*N* = 171)	Patients with NS (*N* = 60)	Patients with simple hematuria (*N* = 28)	Patients with other symptoms (*N* = 83)	*P*
Age (years)	8.86 ± 3.24	7.63 ± 3.39	9.43 ± 3.10	9.44 ± 3.00	<0.01
Male sex, *n* (%)	122 (71.3)	40 (66.7)	20 (71.4)	62 (74.7)	>0.05
Course of illness (months)	5.02 ± 1.14	1.88 ± 0.83	15.71 ± 5.39	3.67 ± 1.19	<0.01
Laboratory measurements					
24 h urinary protein excretion (mg/m^2^)	82.08 ± 24.72	2,629.68 ± 1,363.92	72.48 ± 12.24	629.52 ± 558.0	<0.01
Serum albumin (g/L)	30.65 ± 9.47	18.74 ± 4.10	39.53 ± 3.84	35.96 ± 4.08	<0.01
Total cholesterol (mmol/L)	5.23 ± 2.31	7.71 ± 2.20	3.40 ± 0.47	3.96 ± 1.13	<0.01
eGFR (ml/min/1.73 m^2^)	89.22 ± 27.35	91.22 ± 27.96	127.38 ± 29.85	86.24 ± 26.82	>0.05

*NS* nephrotic syndrome, *eGFR* estimated glomerular filtration rate

### Clinical features of patients with NS according to response of steroid therapy

Among the 60 patients with nephrotic syndrome, 2 patients were excluded from the study because they refused steroid therapy and were treated with traditional Chinese medicine. The remaining 58 patients received 2 mg/kg/day of oral prednisone (maximum dose ≤ 60 mg/day).

Among the 58 patients who received prednisone therapy, 14 patients were in complete remission (steroid-sensitive) in 8 weeks, and 44 patients were not in complete remission (steroid-resistant) in 8 weeks. Among the 14 steroid-sensitive patients, 5 patients (35.7 %) had hematuria, 1 patient with gross hematuria and 4 patients with microscopic hematuria. Among the 44 steroid-resistant patients, 40 cases (90.9 %) had hematuria, 14 patients with gross hematuria and 26 patients with microscopic hematuria. There was a significant difference in the incidence of hematuria, eGFR (106.89 ± 26.94 vs 86.69 ± 26.85 ml/min/1.73 m^2^), and age (5.86 ± 3.11 vs 8.19 ± 3.31 years old) between the steroid-sensitive and the steroid-resistant groups (Table 2). The correlation between response of steroid therapy and morphological variables of the Oxford-–MEST classification are summarized in Table 2. Except for the nonsignificant association of response of steroid therapy with the E variant of the Oxford classification (*p* = 0.318), other variants of the Oxford classification, including M, S, and T morphological variables, had a significant association with response to steroid therapy (M: *p* = 0.001; S: *p* = 0.046; and T: *p* = 0.04).

**Table 2:** Analysis of patients with immunoglobulin A nephropathy (IgAN) and nephrotic syndrome who accepted steroid therapy

	All patients (*N* = 60)	Accepted steroid therapy (*n* = 58)		
		Sensitive to steroid (*n* = 14)	Resistant to steroid (*n* = 44)	*P*
Age (years)	7.63 ± 3.39	5.86 ± 3.11	8.19 ± 3.31	<0.05
Male sex, *n* (%)	40 (66.7)	9 (64.3)	30 (68.2)	>0.05
Hematuria, *n* (%)	47 (78.3)	5 (35.7)	40 (90.9)	<0.01
Gross hematuria, *n* (%)	17 (28.3)	1 (7.1)	14 (31.8)	<0.05
Microscopic hematuria, *n* (%)	30 (50.0)	4 (28.6)	26 (59.1)	>0.05
AKI, *n* (%)	19 (31.7)	2 (14.3)	16 (36.4)	>0.05
Laboratory measurements baseline				
Serum albumin (g/L)	18.74 ± 4.10	16.69 ± 3.94	19.39 ± 3.98	>0.05
Total cholesterol (mmol/L)	7.71 ± 2.20	7.95 ± 2.67	7.64 ± 2.07	>0.05
eGFR (ml/min/1.73 m^2^)	91.22 ± 27.96	106.89 ± 26.94	86.69 ± 26.85	<0.05
Follow-up duration (months)	16.24 ± 11.54	16.31 ± 9.55	16.22 ± 12.22	>0.05
Oxford classification, *n* (%)				
Mesangial hypercellularity				<0.01
M0	40 (66.7)	14 (100)	25 (56.8)	
M1	20 (33.3)	0 (0)	19 (43.2)	
Endocapillary hypercellularity				>0.05
E0	33 (55)	9 (64.3)	23 (52.3)	
E1	27 (45)	5 (35.7)	21 (47.7)	
Segmental glomerulosclerosis				<0.01
S0	35 (58.3)	13 (92.9)	21 (47.7)	
S1	25 (41.7)	1 (7.1)	23 (52.3)	
Tubular atrophy/interstitial fibrosis				<0.05
T0	45 (73.3)	14 (100)	29 (65.9)	
T1	11 (18.3)	0 (0)	11 (25)	
T2	4 (8.3)	0 (0)	4 (9.1)	

*AKI* acute kidney injury, *eGFR* estimated glomerular filtration rate

**Fig. 1:**
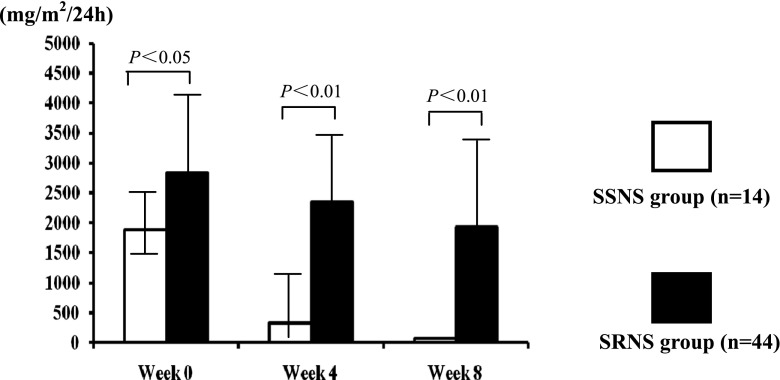
Levels of 24-h urinary protein excretion in steroid-sensitive nephrotic syndrome (*SSNS*) patients and steroid-resistant nephrotic syndrome (*SRNS*) patients (mg/m^2^/24 h) at weeks 0, 4, and 8 of the steroid therapy

The 24-h urinary protein excretion levels in the steroid-sensitive group were 1,885.44 ± 671.69, 322.11 ± 671.16, and 66.10 ± 9.84 mg/m^2^/24 h respectively at weeks 0, 4, and 8. In the steroid-resistant group, the levels were 2,846.36 ± 1,440.06, 2,337.42 ± 1,380.5724, and 1,932.62 ± 1,714.81 mg/m^2^/24 h respectively at weeks 0, 4, and 8. The differences in 24-h urinary protein excretion between the steroid-sensitive and the steroid-resistant groups were significant at all three time points (*P* < 0.05; Fig. 1). The differences among the weeks 0, 4, and 8 in the steroid-sensitive group (*P* < 0.01) and the steroid-resistant group (*P* < 0.05) were also significant (Fig. 1).

### Response of MMF therapy in NS patients with resistance to steroids

Among the 44 steroid-resistant patients, 33 patients were given a combination of MMF and prednisone. Of these 33 patients, 21 patients showed complete remission in 4 months, 6 patients were with partial remission, and 6 patients revealed that the treatment did not evoke any response. The patients were classified into three groups according to their responses to MMF: complete remission (CR), partial remission (PR), and no response (NR) groups. There was no significant difference in age, gender, the incidence of hematuresis, serum albumin, or serum cholesterol among the three groups of patients (Table 3) at the beginning of the study. However, eGFR and the incidence of AKI among patients in the CR group were significantly lower than patients in the PR and NR groups at the beginning of the study (Table 3). The correlation between effect of MMF therapy and morphologic variables of the Oxford-MEST classification are summarized in Table 3. Except for the significant association of effect of MMF therapy with the T variant of the Oxford classification (*p* = 0.001), other variants of the Oxford classification, including M, E and S morphologic variables, had no significant association with effect of MMF therapy (M: *p* = 0.278; E: *p* = 0.686; and S: *p* = 0.113).

**Table 3:** Analysis of immunoglobulin A nephropathy (IgAN) patients who had nephrotic syndrome accepted mycophenolate mofetil (MMF) therapy

	All patients (*N* = 33)	Complete remission (*n* = 21)	Partial remission (*n* = 6)	No response (*n* = 6)	*P*
Age (years)	7.82 ± 3.28	6.86 ± 2.53	9.03 ± 4.60	9.99 ± 3.28	>0.05
Male sex, *n* (%)	22 (66.7)	14 (66.7)	5 (83.3)	3 (50)	>0.05
Hematuria, *n* (%)	31 (93.9)	19 (90.5)	6 (100)	6 (100)	>0.05
Gross hematuria, *n* (%)	11 (33.3)	6 (28.6)	2 (33.3)	3 (50)	>0.05
Microscopic hematuria, *n* (%)	20 (60.60)	13 (61.9)	4 (66.7)	3 (50)	>0.05
AKI, *n* (%)	10 (30.3)	3 (14.3)	3 (50)	4 (66.7)	<0.05
Laboratory measurements baseline					
Serum albumin (g/L)	19.44 ± 4.17	18.82 ± 4.33	20.01 ± 3.00	21.03 ± 4.70	>0.05
Total cholesterol (mmol/L)	7.82 ± 2.04	7.86 ± 2.25	7.52 ± 1.67	7.97 ± 1.85	>0.05
eGFR (ml/min/1.73 m^2^)	92.00 ± 24.98	100.04 ± 18.47	92.24 ± 27.63	72.17 ± 27.55	<0.05
Follow-up duration (months)	18.70 ± 12.41	24.33 ± 12.13	14.00 ± 6.73	13.57 ± 6.61	>0.05
Oxford classification, *n* (%)					
Mesangial hypercellularity					
M0	17 (51.5)	14 (66.7)	3 (50.0)	1 (16.7)	
M1	16 (48.5)	7 (33.3)	3 (50.0)	5 (83.3)	
*P*					>0.05
Endocapillary hypercellularity					
E0	17 (51.5)	12 (57.1)	3 (50.0)	2 (33.3)	
E1	16 (48.5)	9 (42.9)	3 (50.0)	4 (66.7)	
*P*					>0.05
Segmental glomerulosclerosis					
S0	16 (48.5)	13 (61.9)	1 (16.7)	2 (33.3)	
S1	17 (51.5)	8 (38.1)	5 (83.3)	4 (66.7)	
*P*					>0.05
Tubular atrophy/interstitial fibrosis					
T0	22 (66.7)	19 (90.5)	2 (33.3)	1 (16.7)	
T1	9 (27.3)	2 (10.5)	4 (66.7)	3 (50.0)	
T2	2 (6.1)	0 (0)	0 (0)	2 (33.3)	
*P*					<0.01

*AKI* acute kidney injury, *eGFR* estimated glomerular filtration rate

**Fig. 2:**
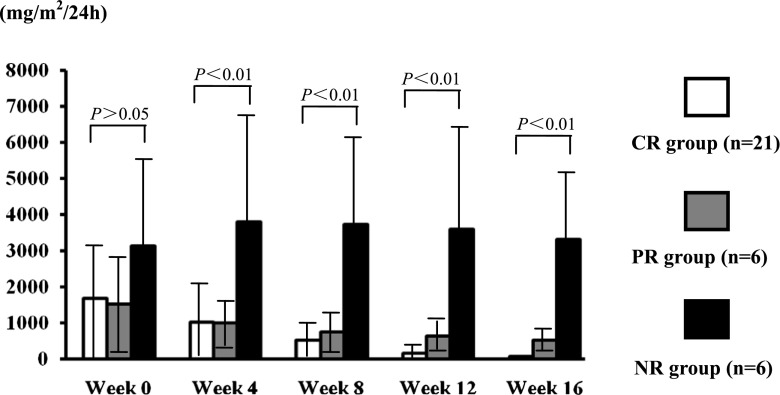
Levels of 24-h urinary protein excretion in complete response (*CR*) patients, partial response (*PR*) patients, and no response (*NR*) patients (mg/m^2^/24 h) at weeks 0, 4, 8, 12, and 16 of the mycophenolate mofetil (MMF) therapy

The 24-h urinary protein excretion levels in the CR group were 1,691.03 ± 1,794.67, 1,029.79 ± 1,040.59, 517.04 ± 467.53, 159.63 ± 114.89, and 74.84 ± 20.43 mg/m^2^/24 h respectively at weeks 0, 4, 8, 12, and 16 of MMF therapy. In the PR group, the levels were 1,517.68 ± 1,415.40, 997.20 ± 717.23, 754.58 ± 544.53, 634.18 ± 433.18, and 517.83 ± 369.48 mg/m^2^/24 h respectively at weeks 0, 4, 8, 12, and 16 of MMF therapy. In the NR group, the levels were 3,126.58 ± 2,360.0, 3,805.24 ± 3,134.49, 3,735.95 ± 2,557.46, 3,591.29 ± 3,513.91, and 3,306.17 ± 2,636.17 mg/m^2^/24 h respectively at weeks 0, 4, 8, 12, and 16 of MMF therapy. The differences in 24-h urinary protein excretion among the CR, PR, and the NR groups were significant at all time points (*P* < 0.05; Fig. 2).

### Follow-up

Among the 14 steroid-sensitive patients, 3 patients were given low-dose prednisone and MMF to maintain remission because of frequency relapse. Four patients who relapsed once or twice are still on low-dose prednisone and maintain remission. For the 7 patients who had stopped prednisone therapy, 6 of them did not experience recurrence and 1 had one relapse.

Among the 21 patients who showed CR to MMF therapy, 18 patients have stopped prednisone and MMF therapy and maintain remission. One patient still has proteinuria after stopping prednisone and MMF therapy for 14 months and was administered traditional Chinese medicine. Stopping therapy 3 months later, 1 patient also had a relapse, while the condition had not improved after receiving combined treatment again. Traditional Chinese medicine therapy was then chosen. One patient relapsed twice during the treatment and then showed MMF resistance, but was relieved after receiving a combination of cyclosporine A (CsA) and prednisone.

Six patients were in PR to MMF therapy, 3 of them stopped prednisone and MMF treatment and were administered traditional Chinese medicine, but their proteinuria did not disappear. Among the remaining 3 patients, 2 had a relapse after receiving CsA and prednisone treatment and are still under maintenance treatment. One patient maintained remission after administration of tacrolimus treatment and has stopped taking medicine for 2 months.

Six patients who received the MMF treatment did not evoke any response, 2 of them showed NR after receiving a combination of tacrolimus and prednisone and are still in follow-up; 4 patients showed no improvement after receiving a combination of CsA and prednisone and were lost to follow-up after choosing traditional Chinese medicine.

Eleven steroid-resistant patients refused MMF treatment and went to other hospitals where they were administered traditional Chinese medicine, but their proteinuria was not reduced after 6 months of treatment. Up to 12 months, 3 patients were lost to follow-up and 3 patients are still under treatment with traditional Chinese medicine. Among the remaining 8 patients, 5 received cyclophosphamide treatment in other hospitals, but the proteinuria did not disappear.

## Discussion

Clinical manifestations of IgAN are various, but dominated by microscopic hematuria or gross hematuria, accompanied by mild to moderate proteinuria, and NS in some children. The data collected from our hospital during December 2005 and May 2013 showed that 33.7 % of children with IgAN have clinical manifestations of NS, which is consistent with the results (30.6 %) reported in a multi-center study in China [18], but higher than that reported for the other countries [17]. This may be because our hospital is a tertiary referral hospital and most referral patients are difficult to treat.

Renal insufficiency, high blood pressure, sustained proteinuria, and nephrotic syndrome are indicators of poor IgAN prognosis [1, 7, 27–29]. In this study, 60 children with IgAN had a clinical manifestation of nephrotic syndrome; patients with hematuria accounted for 78.3 %, and patients with AKI accounted for 31.7 % of all patients. Histologically, mesangial hypercellularity was present in 33.3 % of patients, endothelial hypercellularity in 45.0 %, segmental sclerosis in 41.7 %, and tubular atrophy/interstitial fibrosis in 25.0 % by Oxford classification. The clinical symptoms and pathological changes of IgAN children with nephrotic syndrome were serious. Apparently, they were at a high risk of nephropathy progression.

The treatment of IgAN children with nephrotic syndrome emphasizes the control and elimination of proteinuria, and the drugs for the treatment are mainly divided into two categories, including non-immunosuppressive drugs, such as the renin–angiotensin system inhibitors, calcium antagonists, and fish oil [30], whose clinical effect is not ideal, and immunosuppressants such as corticosteroid and cytotoxic drugs.

The present study tested the efficacy of corticosteroids and MMF on IgAN children with nephrotic syndrome. In the study, 58 IgAN children with nephrotic syndrome received corticosteroid treatment. Among them, 14 patients (24.1 %) were sensitive to steroid therapy, with complete remission of urinary protein, whereas 44 patients showed steroid resistance. The children in the steroid-sensitive group and those in the steroid-resistant group were not significantly different in terms of gender, serum albumin level, hematuria, AKI, or cholesterol level, but they were significantly different with regard to renal pathology grading using the Oxford classification method. In the steroid-sensitive group morphological variables of the Oxford–MEST classification, M, S, and T morphological variables were 0 in most children. In the steroid-resistant group (44 patients), mesangial hypercellularity, segmental sclerosis, and tubular atrophy/interstitial fibrosis were present in most children.

Many researchers believe that steroids can relieve proteinuria in IgAN patients, and slow down the progress of the disease [31–36]. Lai et al. [14] demonstrated that IgAN patients with nephrotic syndrome and mild pathological changes were sensitive to steroid therapy, which had a good effect on these patients. However, these clinical studies were on adults, whereas the present study treated 58 IgAN children who had nephrotic syndrome with steroid therapy. To our knowledge, the sample size in the present study was the largest among similar studies. Our results were similar to those obtained in adults in that patients with minor pathological changes were sensitive to steroid therapy, while those with diffuse mesangial proliferation, segmental glomerulosclerosis, and tubular atrophy/interstitial fibrosis pathological changes were resistant to steroid therapy.

In addition, there was a difference in the clinical characteristics and the epidemiological characteristics between the steroid-sensitive group and the steroid-resistant group. The patients in the steroid-sensitive group were mostly preschool children, while those in the steroid-resistant group were mainly school-aged children. The hematuria incidence, 24-h urinary protein excretion, and eGFR of children in the steroid-sensitive group were significantly lower than those in the steroid-resistant group. Some studies have found that the pathogenesis of IgAN patients with nephrotic syndrome who have a good response to steroid therapy and minor renal pathological changes is different from that of steroid-resistant patients with serious pathological changes; the former consists of minimal lesions of nephrotic syndrome with IgA deposits, whereas the latter consists of real IgAN [37, 38]. However, whether the pathogenesis of the two groups differs is still unclear and needs further exploration.

Mycophenolate mofetil is an immunosuppressant drug that suppresses acute host rejection of renal grafts. MMF is a derivative of the active substance mycophenolic acid. It antagonizes purine metabolism and selectively suppresses T and B lymphocytes dependent on de novo synthesis of purines, thus exerting immunosuppressive effects [39]. Therefore, MMF has little effect on other cells and is unlikely to cause severe adverse reactions such as bone marrow suppression. This drug has been used to treat intractable nephrotic syndrome in children [40–44]. However, the curative effect of MMF in patients with IgAN is controversial [45–48], and, thus, it is not included in the conventional treatment of IgAN. Clinical studies on use of MMF to treat IgAN are currently carried out in adults, and not in children. Patients of children with severe IgAN are uncommon, and most of them have hematuria and mild proteinuria, which can be treated with non-immunosuppressants. The treatment of IgAN children with nephrotic syndrome who are resistant to steroids is rarely reported. We applied combined prednisone and MMF treatment in 33 steroid-resistant IgAN children with nephrotic syndrome, whose renal pathological changes were serious, and found that the therapeutic effect of MMF was dramatic. The proteinuria was found to be in complete remission in 21 patients (63.6 %), partial remission in 6 patients (18.2 %), and no response in 6 patients (18.2 %), which is different from the results of Maes et al. [47] and Frisch et al. [48] who reported that MMF does not affect urinary protein excretion or improve renal function in adult patients with IgAN. The difference may be attributed to the different inclusion criteria of patients. In the studies by Maes et al. and Frisch et al. the authors enrolled adults with low to medium renal function injury, while in our study we recruited children with normal renal function and short disease duration. Furthermore, the factors affecting the efficacy of MMF were analyzed. The results suggested that the T variable of the Oxford–MEST classification and eGFR were the key factors that affected the curative effect of MMF. Most patients (81.8 %) who had the T1 or T2 variable of the Oxford–MEST classification were resistant to MMF treatment, most of those with T0 (86.4 %) were sensitive to MMF treatment, and MMF treatment had a poor effect on those with AKI. The unsatisfactory effect of MMF treatment in adult patients with IgAN might be associated with serious renal pathological damage accompanied by renal impairment.

In summary, this study investigated the curative effect of corticosteroid and MMF on IgAN children with nephrotic syndrome, and demonstrated that the curative effect of corticosteroid and MMF was closely associated with renal pathological damage and renal function. Patients with slight pathological damage, in whom M, S, and T variants of the Oxford classification were 0, had a good response to the steroid treatment, while patients with serious pathological changes were resistant to the drugs. The combined treatment of MMF and prednisone was effective for steroid-resistant patients with renal pathology changes who had T0 of the Oxford–MEST classification, but was poor for those with pathological damage who had T1 or T2 of the Oxford–MEST classification.

However, we were aware of some limitations of this study: it was a single-center study with a small sample size, and it was not a randomized controlled trial, which may have led to bias. Therefore, multi-center studies with large sample sizes are needed to verify these results in the future.
